# Multi-Functional Soft Strain Sensors for Wearable Physiological Monitoring

**DOI:** 10.3390/s18113822

**Published:** 2018-11-08

**Authors:** Josie Hughes, Fumiya Iida

**Affiliations:** Bio-Inspired Robotics Lab, University of Cambridge, Trumpington Street, Cambridge CB2 1PZ, UK; fi224@cam.ac.uk

**Keywords:** soft sensors, wearable sensors, gait detection

## Abstract

Wearable devices which monitor physiological measurements are of significant research interest for a wide number of applications including medicine, entertainment, and wellness monitoring. However, many wearable sensing systems are highly rigid and thus restrict the movement of the wearer, and are not modular or customizable for a specific application. Typically, one sensor is designed to model one physiological indicator which is not a scalable approach. This work aims to address these limitations, by developing soft sensors and including conductive particles into a silicone matrix which allows sheets of soft strain sensors to be developed rapidly using a rapid manufacturing process. By varying the morphology of the sensor sheets and electrode placement the response can be varied. To demonstrate the versatility and range of sensitivity of this base sensing material, two wearable sensors have been developed which show the detection of different physiological parameters. These include a pressure-sensitive insole sensor which can detect ground reaction forces and a strain sensor which can be worn over clothes to allow the measurements of heart rate, breathing rate, and gait.

## 1. Introduction

Obtaining accurate information about a person’s activity and behavior is a key challenge in pervasive computing and has innumerable applications ranging from medicine and rehabilitation to entertainment scenarios [1]. The development of wearable sensors has enabled human activity to be monitored through the accompanying development of low-power, low-cost wireless hardware [2]. The monitoring of physiological signals such as heart rate, respiration rate, and muscle behavior is an area in which wearable sensors can make considerable impact [3]. To achieve physiological measurements, sensors must perform measurements without impeding movement or being obtrusive. The sensors must also show longevity and repeatability which is currently a technological challenge. Therefore, there is need for multi-functional wearable sensors which are compact, lightweight, can detect a range of physiological stimuli, and do not restrict movement [4].

Currently, many wearable sensing techniques use traditional “rigid” sensing materials and only detect one specific physiological stimulus. Many are uncomfortable for the users and restrict or affect their typical movements. Soft sensors have a mechanical impedance closer to that of the wearer, such that the human movement is not impeded. The sensors do not restrict natural kinematics, enabling long-term, comfortable monitoring for the wearer. There has been increasing development of soft sensing wearable devices in areas such as ubiquitous computing and soft robotics, which are gaining increasing traction. However, many existing soft sensors lack the robustness, ease of integration (often full body suits are required) and the flexibility in design [5].

This paper presents a method for manufacturing a soft sensor material which detects deformation resulting from applied force or normal pressure. This sensor can be used to detect a range of different physiological stimuli and uniquely, the approach allows for both rapid production and also the ability to easily change the sensor morphology. The sensor can be used to detect deformations of different magnitudes and profiles with minimal additional circuitry or amplification required. The sensor can undergo strains of over 200% and has a low mechanical impedance so it does not impede movement while also displaying sensitivity to stimuli.

To demonstrate the capability of the material, an insole pressure sensor to detect gait and the ground reaction force has been developed. This insole sensor highlights the ability of the sensor material to detect forces normal to the sensing material. A lateral strain-sensing wireless wearable device has also been developed which allows the breathing rate, heart rate, and calf muscle deformation to be measured by wearing the device at different positions on the body. The results from the strain sensor enable the direction of walk and the gait types to be identified.

**Figure 1:**
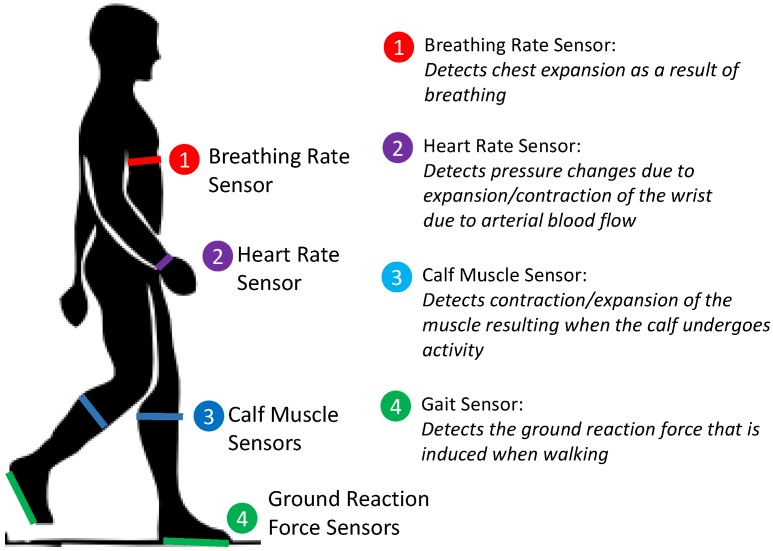
Summary of the different wearable sensors developed, and the locations they should be worn to enable different stimuli to be measured.

The range of different physiological stimuli which can be detected demonstrates the multi-functionality of the sensor. In addition to flexibility, the sensing material is waterproof, easily fabricated and can be worn over clothes. Figure 1 summarizes the different wearable sensors which have been developed to demonstrate the capabilities of the soft sensor.

### 1.1. Motivation for Soft Physiological Sensing

The ability to measure physiological signals on the body accurately and precisely has many applications including sports science, health care, and medicine. The key physiological information includes breathing rate, heart rate, gait, and muscle deformation, all of which can be detected through sensors which measure deformation.

Breathing rate sensors are used widely for medical applications, particularly the identification of heart disease [6] and sports analysis [7,8]. Many of these sensors are bulky, impede movement, and provide low detection sensitivity [9]. Developing a breathing rate sensor which is highly flexible and can be worn over clothes unobtrusively would increase usability.

Typically, heart rate is measured using a rigid finger clip which measures the absorbance of light as the blood pulses through a finger [10]. It may be necessary for heart rate sensors to be worn for extended periods of time. A softer, more flexible alternative would provide greater comfort to the users. There has been some limited development of soft sensors for heart rate monitoring; however, there has been limited successful demonstration [11].

Gait analysis is of interest to researchers and clinicians as it allows the identification of the kinematic parameters of gait which can provide quantitative analysis of muscular-skeletal functions [12]. Such sensors are used in sports to analyze and optimize performance [13], for rehabilitation, to monitor the healing of patients and for health diagnosis to determine muscular-skeletal problems and diseases [14]. Being able to precisely understand the force applied to specific foot locations has many applications. There are existing force sensors used to measure ground reaction forces (GRF) when walking by including sensors in an instrumented shoe. Determining muscle activity and deformation can also be used to determine gait characteristics particularly by considering the deformation of the calf muscle. Strain-detecting sensors can also be used to detect muscle activity such as grasping, or lifting [15], enabling the identification of activity.

### 1.2. Review of Existing Physiological Monitoring Approaches

There are two main approaches for measuring physiological signals: measuring deformation induced by the physical changes in the body or measuring positional change using tri-axial accelerometers and gyroscope-based systems [16,17,18]. The accelerometer-based systems are typically rigid devices with multiple sensors required. Such methods can make it difficult to determine particular muscle behavior or to isolate specific physiological signals.

Some semi-flexible approaches have been developed including Magnetic Field Sensors [19], flexible PCB-based sensors [20] and optical sensors [21]. These are only semi-flexible and often must be in direct contact with the skin. Softer sensors, graphite-based strain sensors [22], and nanowire sensors [23] are available, but these show limited stretchability with limited integration into wearable sensors.

Intelligent textiles such as knitted strain-sensing material have been developed which have the capacity to measure physiological parameters of the human body [22,24]. There are several systems which can be integrated into textiles, to create sensing “suites” for lower limb movement detection [25] or integrating conductive thermoplastic strain sensors into fabrics [26]. These have the advantage of being highly flexible; however, they require the user to wear additional tight clothing and provide only strain sensing. Other wearable sensors rely on the integration of electrodes into the fabric which limits the flexibility [27]. Such sensors often do not have enough sensitivity to measure multiple physiological indicators.

## 2. Materials and Methods

### 2.1. Material Fabrication

The soft sensing material has been developed by including low percentages of conductive particles, Carbon Fiber (CF) and Carbon Black (CB), into a non-conductive matrix. A similar principle has been demonstrated previously, with the inclusion of conductive CB elements in thermoplastics [26]. There has been previous investigation of the integration of CB into a silicon matrix [28,29], the inclusion of other metal-polymers [30] and the inclusion of carbon nanotubes [31]. By including two conductive elements, the amount of each conductive element required is reduced, limiting the effects each has on the mechanical properties of the material. When only CB is included into the silicone, the curing process can inhibit the conductivity of the CB particles due to their low surface area to volume ratio, such that a large percentage weight is required, which significantly affects the material properties of the sensor. Including a small amount of CF (approximately 1% by weight) enables effective conductive pathways to remain after curing, such that lower percentages of CB (again, approximately 1% by weight) are required [32]. Several different theories have been proposed for the electron transport in conductor-filled plastic or rubber systems, the most common and widely accepted is percolation in a continuous conducting network with the additional potential for tunneling between isolated conducting particles [33,34]. The focus of this paper is on the application and usage of the material developed, thus the focus of this paper remains on the application and usage of the sensing material.

To develop the sensing material, the CB and CF particles should first be mixed with a solvent and allowed to dry such that the particles are separated before being included into the silicone mix. In this case, EcoFlex Smooth On 00-20 silicone has been used. The particles should be mixed thoroughly to ensure a homogeneous distribution throughout the mix. The silicone sensor can then be poured into a mold and de-gassed to remove and prevent gas bubbles forming. The silicone sensor is then left to cure, after which the sensor can be released from the mold and cut into the shape required.

**Figure 2:**
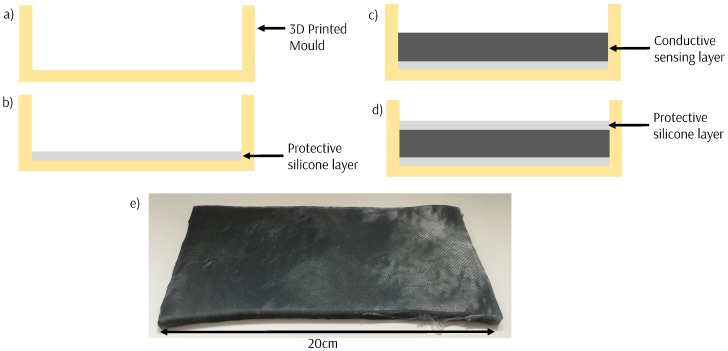
The fabrication process to develop sheets of the sensing material. (**a**) Create a 3D-printed mold for the sensor. (**b**) Cure a thin layer of silicone in the bottom of the mold. (**c**) Create the sensing material and pour into the mold and cure. (**d**) Add a final thin protective layer of silicone. (**e**) The sensing sheets produced.

The silicone sensor material is formed into sheets by sandwiching between two thin insulating outer layers silicone (Figure 2) which are formed in 3D-printed molds. This protects the sensor and allows the sheets to be easily cut into the shape required. The sensors can be made fully waterproof by surrounding them in a full layer of silicone.

The fabrication process is quick (approximately 4 h) and allows for customization of the base resistance and the sensitivity by varying the amounts of CB and CF included and the 3D shape of the sensor developed. The material can be formed into a shape or morphology by 3D printing a mold of a desired shape and allowing the sensor to cure in the mold. Silver-coated electrodes are placed into the sensing material and fixed with adhesive to provide an electrical contact to the sensing material.

### 2.2. Sensor Characterization

**Figure 3:**
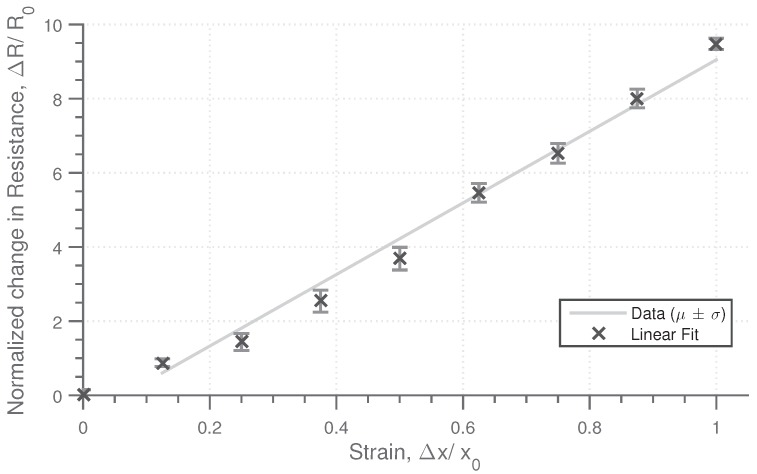
Plot of force against normal strain for the soft conductive silicon sensor showing the mean and standard deviation. Strain was applied in steps of 10%, with this cycle repeated 5 times with the average and standard deviation determined.

The sensing material responds to both lateral and longitudinal strain in addition to normal pressure. The characteristic response of the material to these stimuli has been obtained for a material sample with a composition of 1% CB percentage and 0.8% weight of CF, with the physical dimensions of (w,h,d) 60 mm × 20 mm × 4 mm. The base resistance as measured across the width is approximately 1 kΩ. To determine the strain characteristics, strain was applied in 10% intervals up to 100%, with the resistance measured at each stage. This cycle was repeated five times. The strain resistance profile, Figure 3, shows an approximately linear change in resistance with strain, with a high repeatability. The change in resistance is significant, with the resistance increasing by a factor of 10 when the strain is increased to 100%, demonstrating a high sensitivity. The sensing material is highly flexible and can continue to deform past 100% strain, to the order of 250% strain before the tensile limit of the material is approached.

**Figure 4:**
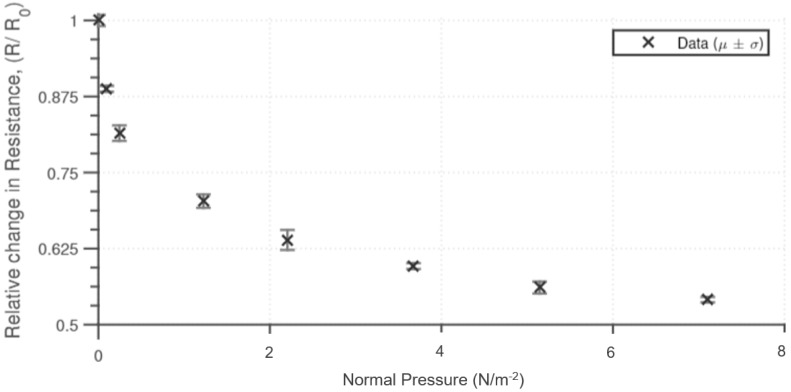
Resistance measured across the sensor (with electrodes placed at either end) when increasing loads (of base diameter 2 cm) are applied to the sensor. The experiment was repeated 5 times with the average and standard deviation shown.

The sensing material is also sensitive to normal pressure. The pressure dependency was determined by applying a normal pressure and measuring the end-to-end resistance of the sensing material. The results (Figure 4) show that for up to 4 Nm^−2^ there is a significant initial reduction in resistance; however, after this point, there is minimal reduction in the resistance measured. A similar response to normal pressure has been obtained as to other similar silicone-based pressure sensors [35]. The material compression properties contribute to the resistive properties of the material, with the rate of resistance decreasing with normal pressure. The range of sensitivity can be altered by changing the physical height of the sensing material and the percentage inclusion of the conductive particles.

### 2.3. Development of Wearable Sensing Devices

#### 2.3.1. Force Detecting Gait Sensor

**Figure 5:**
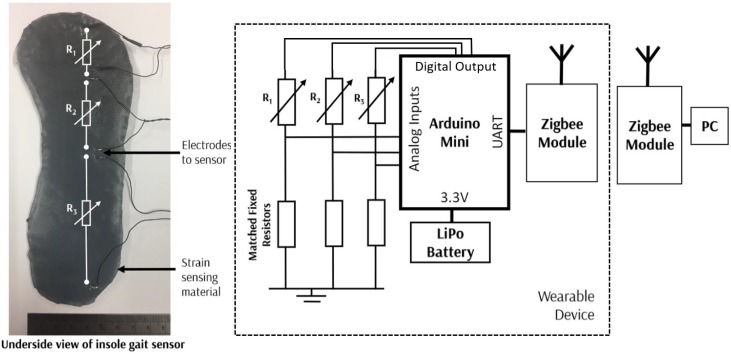
Underside of the soft silicone gait sensor developed showing the attachment to the electrodes (**left**) and the associated circuitry for the system, showing the interface between the sensor and the microcontroller and the wireless system (**right**).

The gait sensor was formed into a shoe insole which measures normal pressure. A sheet of the conductive silicone material was produced and then cut to the required shape to form the insole. Electrodes can then be attached at any points on the sensor, to measure the pressure applied between the connection points. For this sensor, electrodes have been applied to measure the pressure distribution under the toe, ball of the foot, and the heel. The electrodes and the corresponding three regions are shown in Figure 5.

The resistance is measured by the three pairs of electrodes with potential dividers used to provide an analogue input to the microcontroller. To measure the resistance across each of the sensing areas, the corresponding digital input is excited, such that the resistance across only that potential divider is measured. Thus, each of the three resistance is measured rapidly in a sequential manner. The microcontroller is powered by a Lithium Polymer battery, and a ZigBee Module is used to provide wireless communication to enable the device to be worn entirely on person.

The sensors are sampled at 20 Hz, with some averaging and filtering performed on the microcontroller. An IIR (Infinite-Impulse Response) filter is implemented in real time with a cut-off frequency of 2 Hz to eliminate high-frequency noise. This filtered data is then transmitted alongside a time stamp to the PC over ZigBee. On the PC the data is read in as a serial input from the ZigBee wireless module. It is then parsed, and data analysis, namely peak detection, is performed. This is performed close to real time. This acquisition procedure is the same used for all the sensors. Although ZigBee is used initially, should this be developed further, a more energy-efficient wireless protocol would be used.

#### 2.3.2. Strain Sensor: Breathing Rate, Heart Rate and Calf Sensor

**Figure 6:**
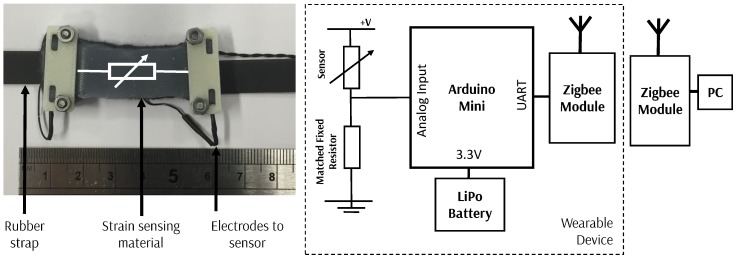
Strain-sensing sensor (**left**) and the overview of the wearable sensing system developed showing the interface between the sensor and the microcontroller (**right**).

A universal wearable sensor to allow the detection of breathing rate, heart rate, and calf muscle behavior has been developed using a similar system to that for the gait sensor (Figure 6). A small section of sensing material is attached to a rubber strap with electrodes added to interface with the microcontroller. To detect breathing rate the wearable sensor can be worn around the chest over clothing, around the wrist to allow detection of heartrate and around the calf (again, worn over clothing) to allow the changes in profile of the calf muscle to be detected. A similar architecture to the gait sensor is used, with a wearable device developed with ZigBee wireless capability.

## 3. Results

### 3.1. Gait Sensor

#### 3.1.1. Experimental Methods

The developed gait sensor was placed inside the shoe with the wireless unit attached to the side of the shoe. Results were obtained from the sensor while two different users were walking each for a five-minute period at a constant rate on a flat surface. In addition, the device was tested when walking, running, and hopping for an approximately five-minute period on a flat surface by one user to allow the accuracy of steps to be determined. To allow comparison to existing calibrated devices, an accelerometer-based step counter device was also worn.

#### 3.1.2. Experimental Results

**Figure 7:**
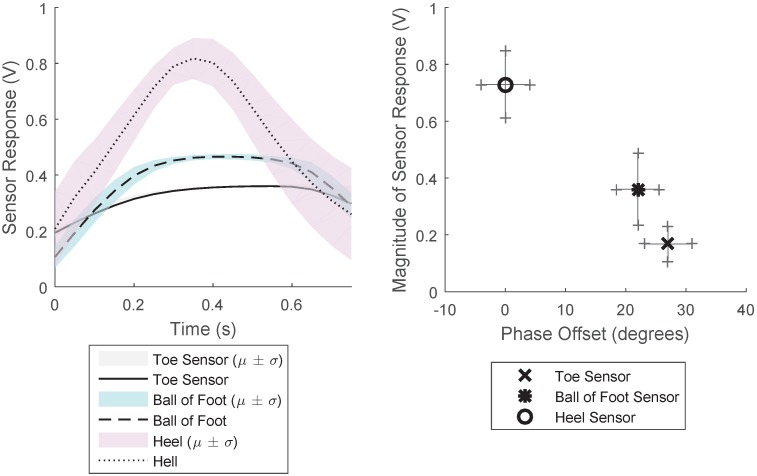
Results from the gait sensor when walking. (**Left**) Average response from the sensor for a single period when walking for a five-minute period for the three sensors at constant walking rate. Found over windowing each period and finding the average and deviation. (**Right**) Plot of magnitude of response of the sensor and phase difference between the responses for a five-minute period of walking at constant walking rate.

The response from the three sections of the gait sensor when worn as an insole of a shoe is shown in Figure 7. This shows the averaged results for a two-minute period for a constant walking rate. This figure shows the average and the standard deviation of the results for this two-minute period, when each period of the response was overlaid. The results show that there is time delay between the pressure detected in the different areas of the sensor. The pressure applied to the heel sensor is significantly larger than that of the other sensors, and the force profile is much sharper than that experienced by the toe and ball of the foot sensor. This provides information as to the gait characteristics: the force profile of the step, the magnitude of the pressure on the specific locations of the sensor and the time difference between the pressure being applied on each part of the sensor. Figure 7 Right shows the phase and magnitude relationship between the three different sensors, demonstrating how the phase and magnitude varies for the three sensors.

To determine the accuracy of the sensor when measuring the number of steps taken, the percentage error when several steps is taken has been determined for different gaits: walking, running, and hopping. The results, Table 1, demonstrate that for fewer than 500 steps the accuracy is high, especially for walking gaits. The accuracy is lowest for running gaits, where the sensor response is noisier. Typical tri-axial accelerometer-based step has a 5% accuracy [36,37], therefore, this gait sensor is comparable or better than traditional “hard” methods of sensing movement. The number of steps taken was calculated by filtering and performing peak detection across all three sensor streams and requiring agreement from two of the three sensors as to the agreement of a “step” having taken place. This led to high accuracy in the detection of steps taken.

### 3.2. Breathing Rate Sensor

#### 3.2.1. Experimental Methods

To experimentally test the sensor, the sensor device was worn around the upper chest over clothes. The sensor was worn when walking, sitting, and running such that a range of different breathing rates and magnitudes of chest expansion were experienced. The sensor data was windowed over a twenty-second period, and the average time between peaks used to determine the breathing rate. A commercial device was also worm simultaneously to provide a ground truth.

#### 3.2.2. Experimental Results

**Figure 8:**
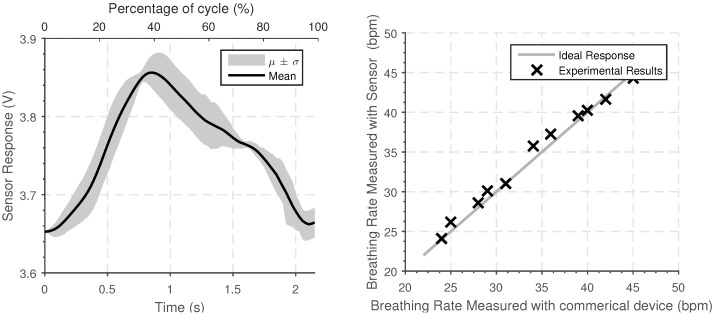
(**Left**) Average sensor response for the sensor when worn around the chest to measure breathing rate for a two-minute period. (**Right**) Experimentally measured breathing rate measured over a thirty-second period and the breathing rate determined by a commercial measurement system (iCare).

A typical sensor response is shown in Figure 8, averaged over four minutes at constant breathing rate. By considering segmenting the segment into different periods of the signal, and overlaying on the rising edge, the average response can be determined. This shows that there is a clear periodic signal that reflects the breathing rate; the magnitude of the response is indicative of the magnitude of the chest expansion. The sensor is sufficiently sensitive that it can be worn over clothing.

To test the sensing system, the results obtained experimentally have been compared to the results from a commercial breathing rate sensor which has a stated accuracy of 1 beat per minute. The results are shown in Figure 8. The device was tested for eight one-minute periods and worn in various different situations (including walking, working, and sitting) with two different users. The results demonstrate that the senor can be used to determine the breathing rate and the magnitude of the chest expansion. Although this only provides initial verification of the system, the variety of conditions tested demonstrates that the system is tolerant to changes in the user’s activity.

### 3.3. Heart Rate Monitor

#### 3.3.1. Experimental Methods

The same sensors used to detect breathing rate can be worn around the wrist to enable the heartrate to be measured. The change in sensor response is much lower than the breathing sensor results as the strain experienced in much less. To ensure that this can be detected without quantization noise of the internal ADC significantly affecting the output reading, an external 12-bit ADC was used.

To test the device, the device was worn on the wrist sufficiently tight such that there are no gaps between the sensor and the wrist. The sensor device was worn when sitting, walking, and running such that different heart rates were experienced. The device was used in typical conditions, as opposed to in a closely controlled lab environment. Two different users were tested with the device. The rolling average time between peaks was used to determine the heartbeat.

#### 3.3.2. Experimental Results

**Figure 9:**
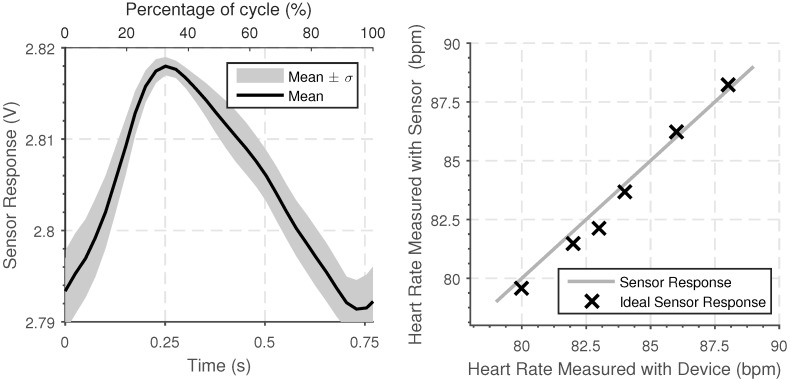
(**Left**) Average time series of the sensor response when the sensor is worn around the wrist for one period of heart beat when measured over a two-minute period. (**Right**) Experimentally measured heart rate measured over a thirty-second period and the heartrate determined by a commercial device (Polar M400, Polar Electro 2018, Finland).

The typical sensor response when the device is worn is shown in Figure 9 Left. This response was again found by overlaying the period of the response, starting at the rising edge, with the average response found.

The accuracy of the heart rate measured using the device was tested by measuring heart rate over a twenty-second period using both the sensing device and using a commercial heart rate device which has a given accuracy of 1 beat per minute. The results were taken when undergoing different activities, including walking, sitting, and working and over a range of different heart rates. The results show agreement between the calibrated commercial device and the wearable sensor developed are shown in Figure 9 Right. The heart rate determined by the sensing device shows a strong agreement with the commercial device, with the maximum different of 1 beat per minute.

### 3.4. Calf Sensors

#### 3.4.1. Experimental Methods

To test the calf sensor, one of the strain sensors was worn on each calf muscle, placed over the location on the calf which experiences maximum deformation. The device was tested when walking, running, and hopping on flat ground for a 5-min period and also when walking in different directions, by recording the response when walking in a circle of constant radius. The device was tested on two users.

#### 3.4.2. Experimental Results

**Figure 10:**
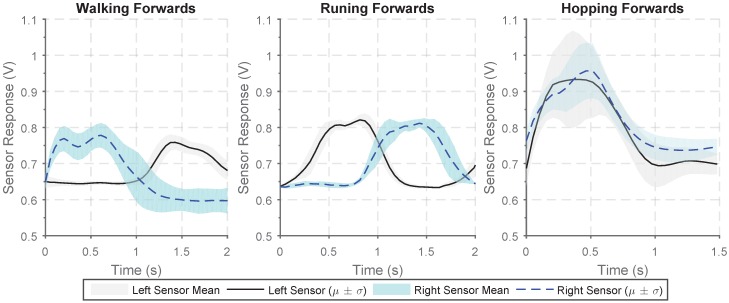
Average sensor output for a single period from two calf strain sensors worn when undertaking various gait types: walking forwards in a straight line, running forwards, hopping forwards.

A sensor can be worn on each calf muscle to allow identification of different gait types and to give an indication of the direction of movement. The sensor output when walking, running, and hopping forwards are shown in Figure 10. There is a clear increase in sensor output from the sensors on the two legs when walking and running with the two sensors response out of phase as the calf muscles in each leg alternatively engage. The frequency of response when running is much greater and the peak-to-peak magnitude of the sensor response is much larger. When hopping, the two sensors response are in phase and there is far more variability in the magnitude of the response.

**Figure 11:**
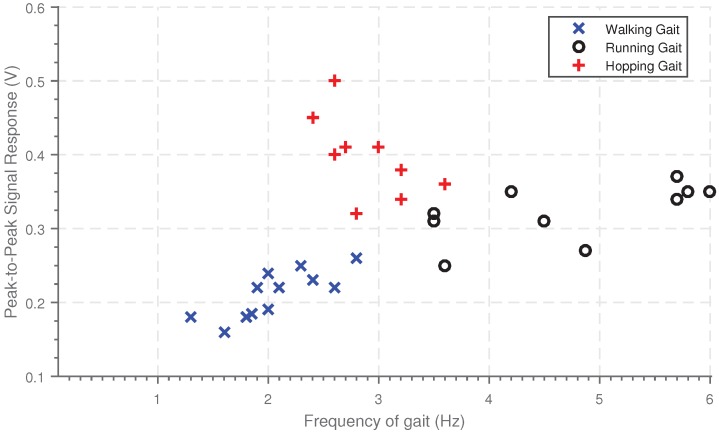
Figure showing the frequency of gait and the peak-to-peak magnitude of response determined from the calf sensors when undergoing various gait types.

Using these sensors to determine the peak-to-peak signal response and the frequency of the gait allows different gait types to be determined. Figure 11 shows the magnitude of the response plotted against frequency of the gait for three different gait patterns. There is clear clustering between the three gait types. Walking has the lowest frequency and magnitude, running has a higher magnitude of sensor response and frequency and finally, hopping has the largest magnitude of response. The phase difference between the two responses could also be used to aid identification of the gain type.

**Figure 12:**
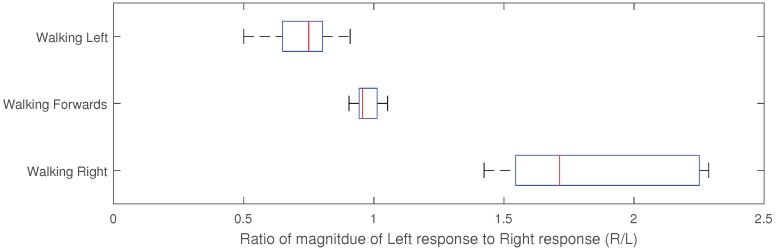
Ratio of the magnitude of the calf response for the Right calf muscle sensor to the left calf muscle sensor for different directions of walk.20 results were recorded for each direction of movement.

By having two calf muscle sensors, it is possible to get an indication of the direction of movement as the expansion and contraction behavior of the two calf muscles varies not walking directly forwards. The magnitude of the average of the peak-to-peak sensor response for the two sensors has been determined for ten-second periods, for 30 different cases of walking left, right and forwards. The ratio of the average magnitude between the right and the left sensor is then found. When walking forwards the sensor response is equal, such that the ratio is approximately 1. When walking right, the left calf muscle activation is greater such that the ratio is greater than one, and conversely when walking left, the right calf muscle is lower such that the ratio is less than one. These results are shown in Figure 12; the different gain types have distinct different ratios allowing unique identification.

## 4. Discussion & Conclusions

This paper presents a sensing material which has been developed by including conductive particles into a non-conductive silicone matrix. This allows the development of sensors in sheets that can be easily cut or formed into the required shape. The sensitivity of the sensor is comparable to existing sensors and can be tuned by varying the CF and carbon powder content added to the silicone. The sensor is reactive to both pressure and strain, allowing it to be used to develop wearable sensors which detect both stimuli.

The pressure-sensing capabilities of the sensing material has been demonstrated through the development of the gait sensor. This sensor enables an indication of the ground reaction force exerted at different locations on the foot to be determined. The gait sensor also enables the approximate frequency, or number of steps taken with comparable accuracy to existing methods and devices. A wearable strain sensor has been developed which can be used to measure breathing rate, heart rate, and calf muscle expansion and contraction. The sensor developed is highly universal such that the same experimental setup can be used to detect these three indicators, which have a range of different magnitudes and frequencies of strain to be measured by the sensor. This demonstrates the high versatility of the sensor. By using pairs of sensors on calf muscles it is possible to provide both an indication of the direction of movement and the type of gait. Initial indications have shown how the sensors can be used to make indications of activities, with gait type and direction of walking already identifiable. By combining all these physiological sensors response, it would be possible to build an overall picture of activity being undertaken.

Although the sample size for testing was low, in this paper we demonstrated the versatility of the sensing material, by testing different sensors on the small sample size. With this sample size, it was possible to identify that the sensors have the capabilities to identify the different physiological signals, the main aim of this paper. However, there was limited assessment of how robust the sensors are to changes in how the sensor is worn. With the sensors developed, the softness and compliance makes them deform to the shape of the wearer and have limited protrusion from skin contact. Therefore, we hypothesize that they are potentially more tolerant to unexpected changes than standard wearable devices.

Further work is required to test each sensor more extensively with a larger testing group to provide greater characterization. This would allow more extensive testing of how sensor is intolerant to changes in the users position, clothing or activity varies.

The sensor developed responds to isotropic strain, allowing the response to be customized by considering placement of the electrodes and modifying the overall morphology. Due to the ease of integration and the potential for low-cost development, this approach has the potential to be used for many other strain-sensing applications. The ability of the sensor to identify both small wrist-based deformations to detect heartrate, yet also large-scale strains experienced during muscle contraction for gait detection, demonstrates the versatility of the sensor. The concept of using a homogeneous sensing material but varying morphology and electrode placement to alter the response provides a new approach to wearable sensor design, where the response is user customizable.

References

## Figures and Tables

**Table 1:** Percentage error of the number of steps taken when using the soft gait sensor for different gait types.

		Movement Type		
	Number of Steps	Walking	Running	Hopping
Percentage Error (%)	200	0%	0%	0%
	500	0.5%	1%	0%
	750	1%	2.5%	2%
